# Hospital-onset *Clostridioides difficile* infections during the COVID-19 pandemic

**DOI:** 10.1017/ice.2020.1223

**Published:** 2020-09-23

**Authors:** Yuying Luo, Lauren T. Grinspan, Yichun Fu, Victoria Adams-Sommer, D. Kyle Willey, Gopi Patel, Ari M. Grinspan

**Affiliations:** 1 The Henry D. Janowitz Division of Gastroenterology, The Icahn School of Medicine at Mount Sinai, New York, New York; 2 Division of Liver Diseases, The Icahn School of Medicine at Mount Sinai, New York, New York; 3 Department of Internal Medicine, The Icahn School of Medicine at Mount Sinai, New York, New York; 4 Division of Infectious Diseases, The Icahn School of Medicine at Mount Sinai, New York, New York

*To the Editor—Clostridioides difficile* infection (CDI) is the most common healthcare-associated infection in the United States.^1^ CDI affects 13 in every 1,000 patients, and ~75% of cases are classified as hospital onset. Antimicrobial stewardship and compliance with hand hygiene and personal protective equipment (PPE) protocols are paramount in efforts to reduce horizontal CDI transmission.^2^ As an epicenter of the coronavirus disease 2019 (COVID-19) caused by severe acute respiratory syndrome coronavirus-2 (SARS-CoV-2), New York City hospitals saw a dramatic increase in admissions and ICU utilization.^3^ To understand the impact of COVID-19 on hospital-onset CDI, we examined antibiotic prescribing patterns, standardized infection ratios (SIRs), and baseline variables in hospitalized adult patients prior to and during the COVID-19 pandemic. We hypothesized that increased antibiotics exposure during the COVID-19 pandemic would lead to a higher incidence of CDI in hospitalized patients.

We conducted a retrospective cohort analysis at a high-volume tertiary-care center comparing a pre–COVID-19 cohort (February–June 2019) of all adult patients who were diagnosed with CDI on admission or during their hospitalization with a cohort during the COVID-19 pandemic (February–June 2020). Baseline categorical variables were compared using χ^2^ tests and continuous variables were compared using the Student *t* test and Mann-Whitney-Wilcoxon test. All analysis was performed in SAS version 9.4 software (SAS Institute, Cary, NC). Primary outcomes of interest included rates of hospital-onset CDI (HO-CDI, defined as a positive *C*. *difficile* test over 3 days after admission),^4^ antibiotic prescribing and length of stay. HO-CDI incidence was described by the standardized infection ratios (SIR), which adjusts for facility and patient-level factors that contribute to hospital-onset infection risk within each facility. Antibiotic prescriptions were measured by antibiotic days per 1,000 days present. The study was approved by the Institutional Review Board of the Icahn School of Medicine at Mount Sinai.

**Fig. 1.**
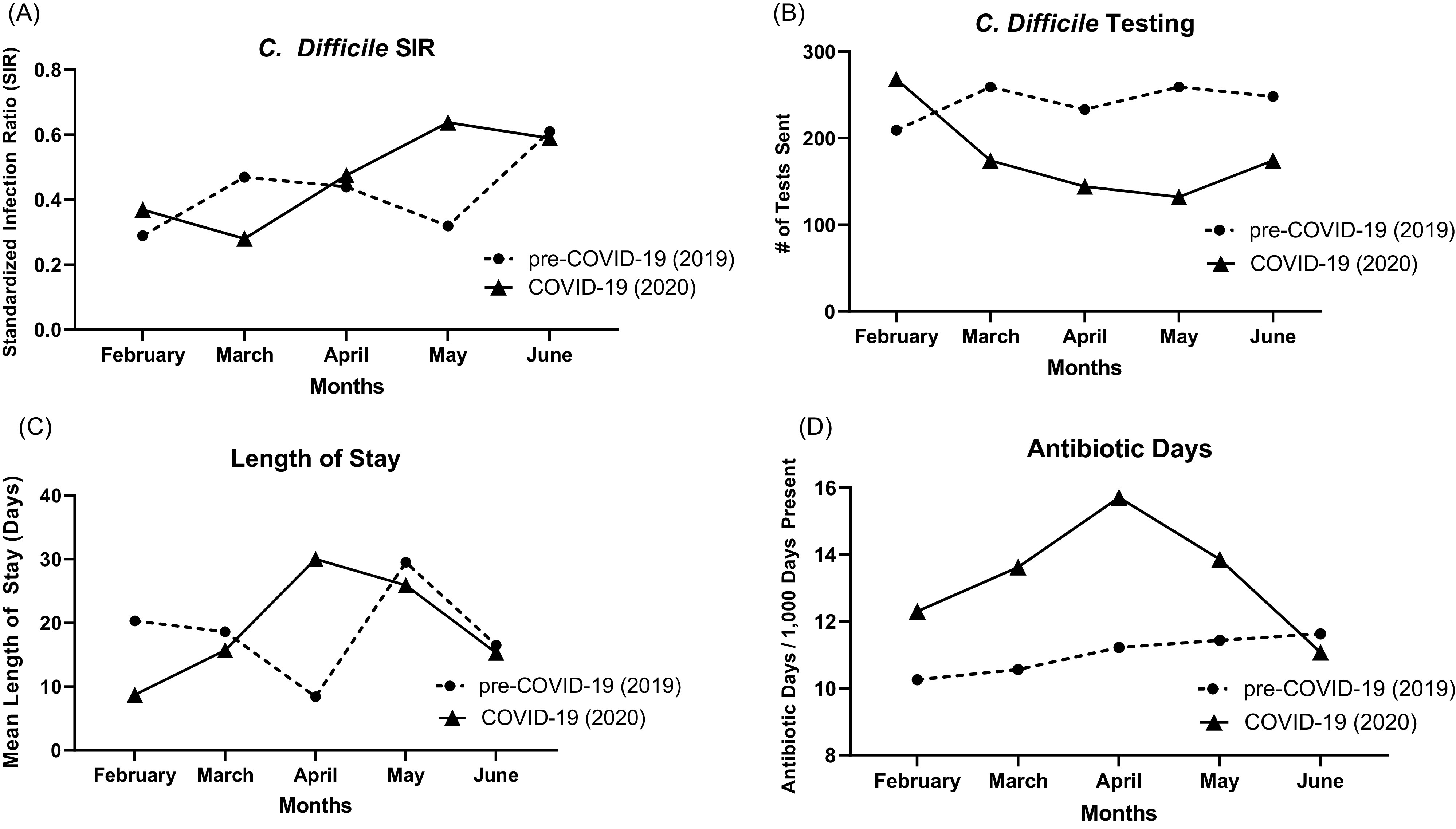
Comparisons between standardized infection ratios (A), *C. difficile* testing (B), length of stay (C) and high-risk antibiotic days (D) between our COVID-19 (2020) and pre-COVID-19 (2019) cohort.

Overall, HO-CDI SIR^5^ was not statistically different during the COVID-19 period than during the 2019 period (*P* = 0.69) (Fig. 1A). For all months, our SIR remained <1, indicating that the number of observed infections was fewer than the number of predicted infections. Compared to the same period in 2019, there were fewer *C. difficile* tests sent during the COVID-19 period, but this was not significant (*P* = .86) (Fig. 1B). Interestingly, we detected a trend toward a higher percentage of positive tests (*P =* .15) during the pandemic than in the pre–COVID-19 time period. We detected a trend toward increased length of stay during the COVID-19 period (Fig. 1C) and increased rate of high-risk antibiotic prescriptions predisposing to CDI, including clindamycin, fluoroquinolones, and third-generation cephalosporins (*P* = .06) (Fig. 1D). There was no difference in mean age at CDI diagnosis, sex, and location at time of CDI diagnosis (eg, intensive care units or stepdown settings versus medical and surgical wards) between the COVID-19 and the pre–COVID-19 cohorts.

At a high-volume, academic, tertiary-care center in an epicenter of the COVID-19 pandemic, we did not find a difference in hospital-onset CDI rate despite a trend toward increased high-risk antibiotic exposures. Although there is growing concern over the increased use of broad-spectrum antibiotics for patients during the pandemic, our data suggest that the rate of CDI was not affected.^6^ We detected a trend toward increased length of stay, especially during our peak COVID-19 census in April, which may predispose patients to hospital-acquired infections, including CDI. We detected a trend toward decreased *C. difficile* testing volume during the COVID-19 period, but a higher percentage of tests returned positive. Patients who presented with diarrhea during the pandemic may have had their diarrheal symptoms attributed to SARS-CoV-2, and *C. difficile* testing may not have been sent in that setting. Although diarrhea can be a symptom of COVID-19, clinicians must be cognizant that these patients remain at high risk for CDI. Our data underscore the continued incidence of HO-CDI in hospitals.

The limitations of our study include lack of patient-level data; individual risk factors for developing HO-CDI (eg, a patient’s immunocompromised status) may have differed between our pre–COVID-19 and COVID-19 cohort. Whether COVID-19 itself increases an individual’s risk for CDI remains unclear. Multiple contributing factors drive CDI incidence, severity, and recurrence. Although PPE use including gowns and gloves during COVID-19 increased, efforts to curb CDI transmission in the hospital setting should continue to emphasize the importance of antimicrobial stewardship, especially as this pandemic re-emerges globally. Reassuringly, CDI rates do not appear to significantly increase during the COVID-19 pandemic.
